# Bovine Milk‐Derived Extracellular Vesicles Inhibit Catabolic and Inflammatory Processes in Cartilage from Osteoarthritis Patients

**DOI:** 10.1002/mnfr.202100764

**Published:** 2022-01-13

**Authors:** Bartijn C. H. Pieters, Onno J. Arntz, Joyce Aarts, Anouk L. Feitsma, R. J. Joost van Neerven, Peter M. van der Kraan, Marina C. Oliveira, Fons A. J. van de Loo

**Affiliations:** ^1^ Department of Rheumatology Radboud University Medical Center Nijmegen Netherlands; ^2^ FrieslandCampina Amersfoort The Netherlands; ^3^ Cell Biology and Immunology Wageningen University & Research Wageningen the Netherlands; ^4^ Department of Nutrition Nursing School Universidade Federal de Minas Gerais Belo Horizonte Minas Gerais Brazil

**Keywords:** cartilage, extracellular vesicles, milk, miR‐148a, osteoarthritis

## Abstract

**Scope:**

Data from the Osteoarthritis Initiative shows that females who drink milk regularly have less joint cartilage loss and OA progression, but the biologic mechanism is unclear. Bovine milk is a rich source of extracellular vesicles (EVs), which are small phospholipid bilayer bound structures that facilitate intercellular communication. In this study, the authors aim to evaluate whether these EVs may have the capacity to protect cartilage from osteoarthritis patients, ex vivo, by directly effecting chondrocytes.

**Methods and Results:**

Human cartilage explants are exposed to cow's milk‐derived EVs (CMEVs), which results in reduced sulfated glycosaminoglycan release and inhibition of metalloproteinase‐1 expression. Incubation of articular chondrocytes with CMEVs also effectively reduces expression of cartilage destructive enzymes (ADAMTS5, MMPs), which play key roles in the disease progression. In part, these findings are attributed to the presence of TGFβ on these vesicles, and in addition, a possible role is reserved for miR‐148a, which is functionally transferred by CMEVs.

**Conclusion:**

These findings highlight the therapeutic potential of local CMEV delivery in osteoarthritic joints, where inflammatory and catabolic mediators are responsible for joint pathology. CMEVs are carriers of both TGFβ and miR‐148a, two essential regulators for maintaining chondrocyte homeostasis and protection against cartilage destruction.

## Graphical abstract

The authors evaluate the effects of extracellular vesicles isolated from commercially available cow's milk on cartilage from osteoarthritis patients. They also found that these vesicles help protect the cartilage from excessive breakdown by limiting the expression of a number of catabolic enzymes. Also, they believe that growth factors and genetic modulators carried by these vesicles are, in part, responsible for these protective effects.

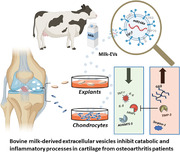

## 1 Introduction

Dairy consumption has long been a topic of debate when it comes to health, and in particular, bone and joint‐related diseases. Numerous studies have shown both beneficial,^[^ ^1^, ^2^, ^3^ ^]^ as well as adverse effects of milk consumption,^[^ ^4^, ^5^ ^]^ depending on the amount and type of dairy product. For osteoarthritis (OA), an age‐related musculoskeletal disease, most studies show a beneficial relationship between consumption of milk and disease prevention.^[^ ^6^, ^7^ ^]^ In the 60s, the consensus was that milk promotes bone growth and density, either as a source of calcium or via enhancing intestinal calcium uptake (reviewed in^[^ ^8^ ^]^). Nowadays, the biological mechanism underlying the protective effects of milk consumption on the progression of OA is not understood at present.

Milk is a complex biological fluid with unique bioactive components, which have an effect on gut immunity, regulation of the intestinal flora, growth and development of infants.^[^ ^9^, ^10^ ^]^ Many proteins, such as lactoferrin, lactadherin, and immunoglobulins are suggested to mediate these effects. Milk also contains an abundance of growth factors, with transforming growth factor beta (TGFβ) being one of the most predominant.^[^ ^11^ ^]^ TGFβ is a key factor in cartilage development and homeostasis, and dysregulated signaling is associated with OA.^[^ ^12^, ^13^ ^]^

Moreover, milk is a rich source of extracellular vesicles (EVs), cell membrane‐derived phospholipid bilayer nanostructures.^[^ ^14^ ^]^ EVs were previously considered to be cellular waste products, but have since been implicated as important mediators in many physiological processes.^[^ ^15^ ^]^ By transporting their cargo, consisting of bioactive proteins, enzymes, and lipids they function as vehicles for intercellular communication. Due to their resilient lipid bilayer membrane, their cargo is protected from degradation, such as low pH and rich enzymatic environments as seen in the gastrointestinal tract.^[^ ^16^, ^17^ ^]^ Milk‐derived EVs also contain an abundance of microRNAs, which have been implicated in immune‐related functions and cellular growth (reviewed in ^[^ ^18^ ^]^). A large part of these highly abundant microRNAs are evolutionary conserved and are found in EVs from multiple species, including human, porcine, and bovine milk.^[^ ^19^ ^]^

Bovine milk‐derived EVs obtained from both raw and pasteurized milk have been characterized in great detail, including their protein and microRNA cargo.^[^ ^20^, ^21^ ^]^ We and others have also shown the bioactivity of bovine milk‐derived EVs obtained from commercially available pasteurized milk, on various cell types. Nordgren et al.^[^ ^22^ ^]^ have shown that in combination with agricultural dust exposure bovine milk‐derived EVs can promote M1 macrophage polarization and we ^[^ ^23^, ^24^ ^]^ found that osteoclasts and osteoblasts differentiation is also modulated by milk EVs. Osteoclast precursor cells differentiated in the presence of bovine milk‐derived EVs showed reduced bone resorbing capacity, whereas osteogenic differentiation from mesenchymal stem cells was accelerated by milk‐derived EVs. Additionally, we found that bovine milk‐derived EVs can ameliorate experimental arthritis and diminish cartilage pathology in mice.^[^ ^25^ ^]^

Glycosaminoglycans (GAGs) are important polysaccharides present in cartilage with the function of shock absorbers in joints. Loss of GAGs is positively correlated to OA progression.^[^ ^26^ ^]^ Currently, there is no treatment to halt progression of OA, and patients rely on knee replacements at end stage as their only therapeutic intervention, as articular cartilage has a limited capacity to repair itself. The loss of GAGs is shown to be a significant factor in these earlier phases of OA development. The cartilage (matrix) quality can be measured by estimating the amount of GAGs in the cartilage of patients, using techniques such as Delayed Gadolinium‐Enhanced Magnetic Resonance Imaging of Cartilage.^[^ ^27^ ^]^

Recent studies have shown the potential of mesenchymal stem cell‐derived EVs to contribute to cartilage repair and protect cartilage from degradation.^[^ ^28^, ^29^ ^]^ If bovine milk‐derived EVs would have a similar effect on cartilage, it would be a cheap and easily accessible source for therapeutic vesicles.

In this study, we investigated the functional effects of bovine milk‐derived EVs on human cartilage as a potential strategy to maintain joint health in healthy individuals and possibly OA patients. We found that exposure to milk EVs resulted in reduced glycosaminoglycan release by cartilage explants, obtained from OA patients. To further investigate this, we studied the effect of milk EVs on human chondrocytes and found that expression of several cartilage degrading enzymes was reduced. Finally, we attempted to unravel the mechanism behind these observations, which in part could be attributed to the presence of TGFβ and miRNA‐148a in milk EVs.

## 2 Experimental Section

### 2.1 Isolation of Milk‐Derived Extracellular Vesicles

Commercial pasteurized skimmed cow's milk was purchased at the local supermarket and stored at 4°C until EV isolation (max one day storage). Milk samples were centrifuged at 70,000 × *g* for 60 min at 4°C, to remove milk fat globules, residual milk fat globules, casein proteins, and other debris (Polypropylene tubes, Thermo Fisher AH‐629 rotor ‐ Max acceleration, no break). The clear supernatant was subsequently filtered through Whatman papers, first #1 and thereafter #50, and finally through 0.45 and 0.2 µm syringe filters. EVs were isolated from the filtered supernatant by ultracentrifugation at 110,000 × *g* for 90 min at 4°C without breaks. EVs, located on top of a firm casein pellet, were washed and taken up in PBS or in the appropriate buffer for RNA or protein analysis. For each isolation roughly 40 mL of milk was processed, resulting in 1 mL of EVs (protein‐particle ratio 5—15 fg particle^‐1^). After isolation, EVs were aliquoted and stored at 4°C for up to 6 weeks. The amount of protein for each isolation was measured with a Micro‐BCA kit (Thermo Scientific, Pierce, Rockford, USA). Further details on the isolation procedure can be found in the Table S1 (Supporting Information), where the minimal information for studies of extracellular vesicles (MISEV2018 ^[^ ^30^ ^]^) checklist is presented.

**Table 1:** Primer sequences

Gene	Forward primer	Reverse primer
ADAMTS‐5	GCTCACGAAATCGGACATTTACTT	ACCAAAGGTCTCTTCACAGAATTTG
IL‐6	AGCCCACCGGGAACGA	GGACCGAAGGCGCTTGT
IL‐8	AGAAGTTTTTGAAGAGGGCTGAGA	CAGACCCACACAATACATGAAGTG
DNMT1	CAGAAAAGGAATGTGTGAAGGAGAA	AAGGGATTTGACTTTAGCCAGGTA
MMP1	ACTGCCAAATGGGCTTGAAG	TTCCCTTTGAAAAACCGGACTT
MMP3	GAGGCATCCACACCCTAGGTT	TCAGAAATGGCTGCATCGATT
MMP13	ATTAAGGAGCATGGCGACTTCT	CCCAGGAGGAAAAGCATGAG
TNFα	TCTTCTCGAACCCCGAGTGA	CCTCTGATGGCACCACCAG
TIMP3	TCTCCTTGTCCCTGCTTCATG	AGGCCCTTGACTACACTCTCATCT
GAPDH	ATCTTCTTTTGCGTCGCCAG	TTCCCCATGGTGTCTGAGC
RPS27A	TGGCTGTCCTGAAATATTATAAGGT	CCCCAGCACCACATTCATCA

### 2.2 Nanoparticle Tracking Analysis

Vesicle size distribution and concentration were estimated by the Brownian motion of particles under constant flow in a NanoSight NS300 equipped with a syringe pump (Malvern Panalytics, Malvern, UK). Concentrations were calculated using Nanoparticle Tracking Analysis 3.2 software (Nanosight Ltd, Amesbury, UK). Vesicles were diluted in PBS, till a suitable concentration for analysis was reached (20‐60 particles per frame). Each sample was measured for 60 s, using the following software settings: flow rate 50, camera level 10, and detection threshold 5. Particle concentration was evaluated for the particles between 30 and 200 nm in diameter.

### 2.3 Western Blot

A minimum of 1.0 × 10^10^ vesicles was used for western blot. Crude milk EVs were digested with RIPA buffer (50 mM Tris‐HCl, pH 7.5, 150 mM NaCl, 1% v/v Nonidet P‐40, 0.5% v/v sodium deoxycholate, and 0.1% SDS) supplemented with protease inhibitor cocktail (Sigma‐Aldrich, St. Louis, Missouri, USA). After heating for 5 min by 95°C, pEV lysates were centrifuged for 20 min at 12,000 × *g*, and supernatant was collected. Samples were loaded on a 10–12% SDS‐PAGE gel and was subjected to electrophoresis at 80 V for 30 min, thereafter 1 h at 100 V at 4°C. Proteins were blotted onto nitrocellulose membranes (on ice, 275 mA, 120 min) and blots were blocked in 5% BSA (>95% purity). Overnight incubation at 4°C with primary antibody was followed by 1 h incubation at room temperature with an HRP‐secondary antibody. Blots were visualized using enhanced chemiluminescence on the ImageQuant LAS400. Antibodies used; CD81 (B‐11), Santa Cruz, sc‐166029; HSP70 (3A3), Santa Cruz, sc‐32239: CD63 (H5C6), BD Pharmingen, 556019; ALIX (1A12), Santa Cruz, sc‐53540.

### 2.4 Electron Microscopy

Milk‐derived EVs were layered on top of a 30% sucrose cushion, and centrifuged for 70 min at 100,000 × *g*, to further purify the EVs. After the sucrose cushion, the EV fraction was subjected to an additional ultracentrifugation step to pellet the EVs. EVs were taken up in small volumes of deionized water, which were placed on nickel grids and allowed to dry for 45 min. The grids with EVs were then washed by transferring them onto several drops of deionized water. Negative contrast staining was performed by incubating the grids on top of drops of 6% uranyl acetate. Excess fluid was removed, and the grids were allowed to dry before examination on a Jeol JEM1400 Transmission Electron Microscope (Jeol, The Netherlands).

### 2.5 Tissue Acquisition, Human Cartilage Explants, and Cell Cultures

Human cartilage was obtained from four anonymous OA donors undergoing total knee replacement. From each individual donor cartilage explants were isolated from macroscopically unaffected regions with a biopsy punch (3 mm diameter) (Kai Medical, Japan). In total, 45 biopsy punches were collected per individual and randomly distributed over the five tested conditions (triplicates, three punches per well). Per condition, the three explants were transferred to a well of a 24‐well plate and rested overnight at 37°C, 5% CO_2_, and 95% humidity in 1 mL DMEM/Ham's F12‐medium (1:1), supplemented with 10% fetal calf serum, 100 mg L^–1^ sodium pyruvate, 100 U mL^–1^ penicillin, and 100 µg mL^–1^ streptomycin before stimulation with milk EVs. Patients were informed about the anonymized use of this material and were able to refuse the use of their material for research. According to Dutch law, informed consent is therefore not necessary.

G6 chondrocytes are heat‐labile SV40 large T oncogene immortalized cells generated in our department, originating from hip‐cartilage from an OA patient.^[^ ^31^ ^]^ Chondrocytes were cultured in HAM‐F12 medium, containing 5% FCS. For stimulation experiments, 100,000 cells were seeded in 24‐well plates (1 mL medium per well).

Primary human chondrocytes were isolated from cartilage slices obtained from anonymous OA donors, by overnight digestion with 2 mg mL^–1^ collagenase B (Roche Diagnostics, Mannheim, Germany). Chondrocytes were seeded at 100,000 cells cm^‐2^ in a 24‐well culture plate (Cellstar; Greiner Bio‐one, Alphen aan de Rijn, the Netherlands). For recovering, chondrocytes were cultured 1 week in DMEM/Ham's F12 (1:1), supplemented with 10% fetal calf serum, 100 mg L^–1^ sodium pyruvate, 100 U mL^–1^ penicillin, and 100 µg mL^–1^ streptomycin in standard conditions (5% CO_2_ (v/v), 37 °C, 95% humidity). Before stimulation experiments, chondrocytes were serum starved for 24 h.

### 2.6 Sulfated Glycosaminoglycans (sGAGs) Measurement

To measure the concentration of released sGAGs from the cartilage explants in the culture medium, the 1,9‐dimethylmethylene blue (DMMB) assay was used. The sGAG content was measured by adding 200 µL DMMB solution (0.05 mM DMMB, 41 mM NaCl, 45 mM glycine, and pH = 3.0) to 40 µL supernatant sample (two, four, and eight times pre‐diluted in water) in a 96‐well plate. The absorbance was measured at 595 nm using an iMark Reader (Bio‐Rad, California, USA).

### 2.7 RNA Isolation and Quantitative RT‐PCR

mRNA was isolated from cells using TRI‐reagent (Sigma‐Aldrich) as described in the manufacturer's protocol. mRNA was treated with 1 µL DNAse (Life Technologies, USA) for 15 min at room temperature and incubated with 1 µL 25mM EDTA (Life Technologies, USA) for 10 min at 65°C. mRNA was reverse transcribed to complementary DNA using 1.9 µL ultrapure water, 2.4 µL 10 × DNAse buffer, 2.0 µL 0.1M dithiothreitol, 0.8 µL 25mM dNTP, 0.4 µg oligo dT primer, 1 µL 200 U µL^–1^ M‐MLV reverse transcriptase (Life Technologies, USA), and 0.5 µL 40U µL^–1^ RNAsin (Promega, The Netherlands) in a single‐step reverse transcription: 5 min at 25°C, 60 min at 39°C, and 5 min at 95°C. Quantitative PCR was performed using SYBR green master mix (Applied Biosystems) with 0.25 mM primers, with the following amplification protocol: 10 min at 95°C, followed by 40 cycles of 15 s at 95°C and 1 min at 60 °C. Melting curves were constructed to determine the specificity of the product. To calculate the relative gene expression, reference genes glyceraldehyde 3‐phosphate dehydrogenase (*GAPDH*) and ribosomal protein S27A (*RPS27A*) were used because isolated RNA levels were significant correlated (data not shown). Primer sequences are listed in **Table** **1**.

### 2.8 Antibody‐Based Capture Assay

Wells of high‐binding microtiter plates were coated with 1 µg of αTGFβ‐1,2,3 antibody (R&D Systems, MAB1835‐500) overnight at 4°C. After washing the plates, CMEVs were added and incubated for 2 h at room temperature. Unbound fractions, TGFβ‐depleted, were used for in vitro experiments.

### 2.9 miR‐148a‐148aT Reporter Assay

Transient transfections were performed in chondrocyte G6 cell line, as well as A549 cells. Twenty‐four hours after seeding in a six‐multiwell plate, cells were transfected using lipofectamine according to the manufacturer's protocol. After 24 h, cells were transferred into 96‐multiwell plates and stimulated with CMEVs for up to 72 h. Thereafter, cells were lysed in water and luciferase activity was quantified using Bright‐Glo luciferase assay system (Promega) by adding an equal volume of Bright‐Glo to the cell lysate. Luminescence was quantified in a luminometer (Clariostar). pluc‐miR148a‐148aT was a gift from Cristina Fillat (Addgene plasmid #65053; http://n2t.net/addgene:65053 ; RRID:Addgene_65053).^[^ ^32^ ^]^

### 2.10 Statistical Analysis

All data are expressed as the mean ± SD or mean ± SEM. Data were analyzed for statistical differences using Mann‐Whitney *U* test. Values of *p* < 0.05 were considered to indicate statistical significance. All statistical analyses were performed using GraphPad Prism 5.01 (GraphPad Software, La Jolla, California, USA).

## 3 Results

### 3.1 Characterization of Extracellular Vesicles Isolated from Commercial Skimmed Milk

**Figure 1:**
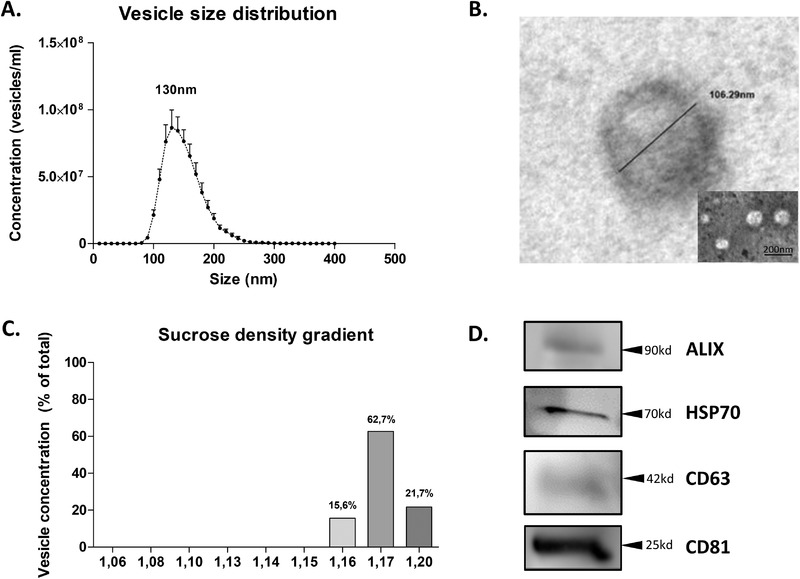
Characterization of commercial milk‐derived extracellular vesicles. Within 2 h, EVs were isolated using ultracentrifugation. A) Particle size distribution of isolated vesicles was determined using a NS300. Data presented is a combination of eight separate isolations, error bars represent mean ± SEM. B) Electron microscopy confirmed spherical morphology and biolayer membrane structure. C) Sucrose density gradient following standard ultracentrifugation‐based isolation shows particles in the range of 1.16–1.20 g mL^–1^, which is the described range for exosome‐like vesicles. D) Western blotting confirmed the presence of EV‐markers ALIX, HSP70, CD63, and CD81.

Bovine milk is a rich source of extracellular vesicles, and it is known that these vesicles can survive processing. Via differential ultracentrifugation, up to 8.4 × 10^9^ particles per milliliter of pasteurized milk ((4.0±0.9) × 10^9^, average ±SEM, *N* = 8) were isolated, with a mean particle diameter of 130 nm [90% CI = 110–200nm] (**Figure** **1**A), which matches the size described for exosome‐like vesicles.^[^ ^20^ ^]^ For each isolation the protein concentration was measured using microBCA. Isolates with a protein concentration between 5–15 ng per 1.0 × 10^6^ particles (9.7±0.9 ng, average ±SEM, *N* = 8) were used for functional studies. Vesicular structure of purified exosomes‐like vesicles is shown in Figure 1B. Besides exosome‐like vesicles, pasteurized milk also contains an abundance of other small particles, such as fat globules and casein micelles.^[^ ^33^ ^]^ To ensure our isolations contain primarily exosome‐like vesicles a sucrose‐based density gradient (top down), which can separate exosome‐like vesicles with a density between 1.15 and 1.21 g mL^–1^ sucrose, from fat globules and casein micelles, which have a lower floating density, was performed. The majority of the vesicles (>98%) ended up within the floating density range described for exosome‐like vesicles (Figure 1C). Next, western blot analysis for four classical EV‐markers was performed for HSP70, CD81, CD63, and ALIX to confirm the purity of the particles in the isolate (Figure 1D).

### 3.2 Bovine Milk Extracellular Vesicles Decrease sGAG release from OA Cartilage Explants

**Figure 2:**
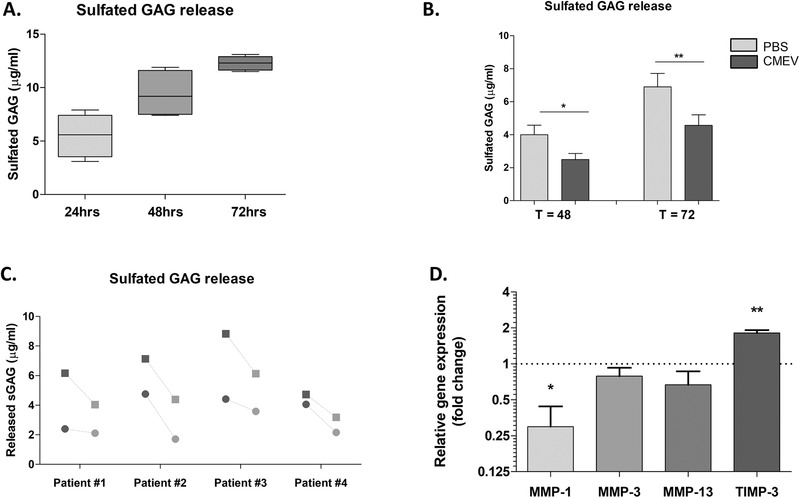
Milk‐derived EVs reduced sGAG release and catabolic gene expression in OA‐cartilage explants ex vivo. Cartilage explants were made from cartilage obtained from OA patients undergoing total knee replacements. In an attempt to minimize the variation between donors, intact cartilage (macroscopically) was chosen from the knee replacement material. A) Quantification of sGAG released into the culture supernatant over 72 h. B) CMEVs reduce the sGAG release, presented as Δ increase compared to T = 24 h (before addition of stimuli). Averages of four patients is shown. C) Individual patients plotted, circles are T = 24 h, squares are T = 48 h, dark grey are PBS controls, light gray are CMEV‐stimulated explants. D) Gene expression analysis for *MMP‐1, MMP‐3, MMP‐13*, and *TIMP‐3*, measured at T = 72 h, plotted as fold change of CMEVs over PBS control. Error bars represent mean ±SEM (N = 4 donors), * *p > 0.05*, ** *p>0.005*.

To study the effect of bovine milk‐derived EVs on cartilage, human cartilage explants obtained from OA patients undergoing total knee replacement surgery were used. First, the loss of sulfated GAG (sGAG) from human OA cartilage explants was investigated. Over the course of three days, a linear increase of sGAG in the culture supernatant (**Figure** **2**A) was observed. To investigate if bovine milk‐derived EVs could protect human cartilage from excessive sGAG release, explants were incubated, starting from day one, with 100 µg mL^–1^ EVs (±1.0 × 10^10^ vesicles). After 24 and 48 h, a significant inhibition of sGAG release (39% and 34%, respectively) compared to PBS control (Figure 2B) was found. As shown in Figure 2C, there was inhibition for two out of four patients at 24 h and for all individual patients at 48 h incubation. Next, gene expression analysis was performed and showed a significant reduction in metalloproteinase‐1 (*MMP‐1*) expression in the CMEV treated group, as well as a significant increase in tissue inhibitor of metalloproteinases (*TIMP‐3*) expression (fold change 0.30 and 1.81, respectively). Expression for *MMP‐3* and *MMP‐13* were not significantly different (Figure 2D).

### 3.3 Milk EVs have a Chondroprotective Effect on OA Cartilage‐Derived Chondrocytes

**Figure 3:**
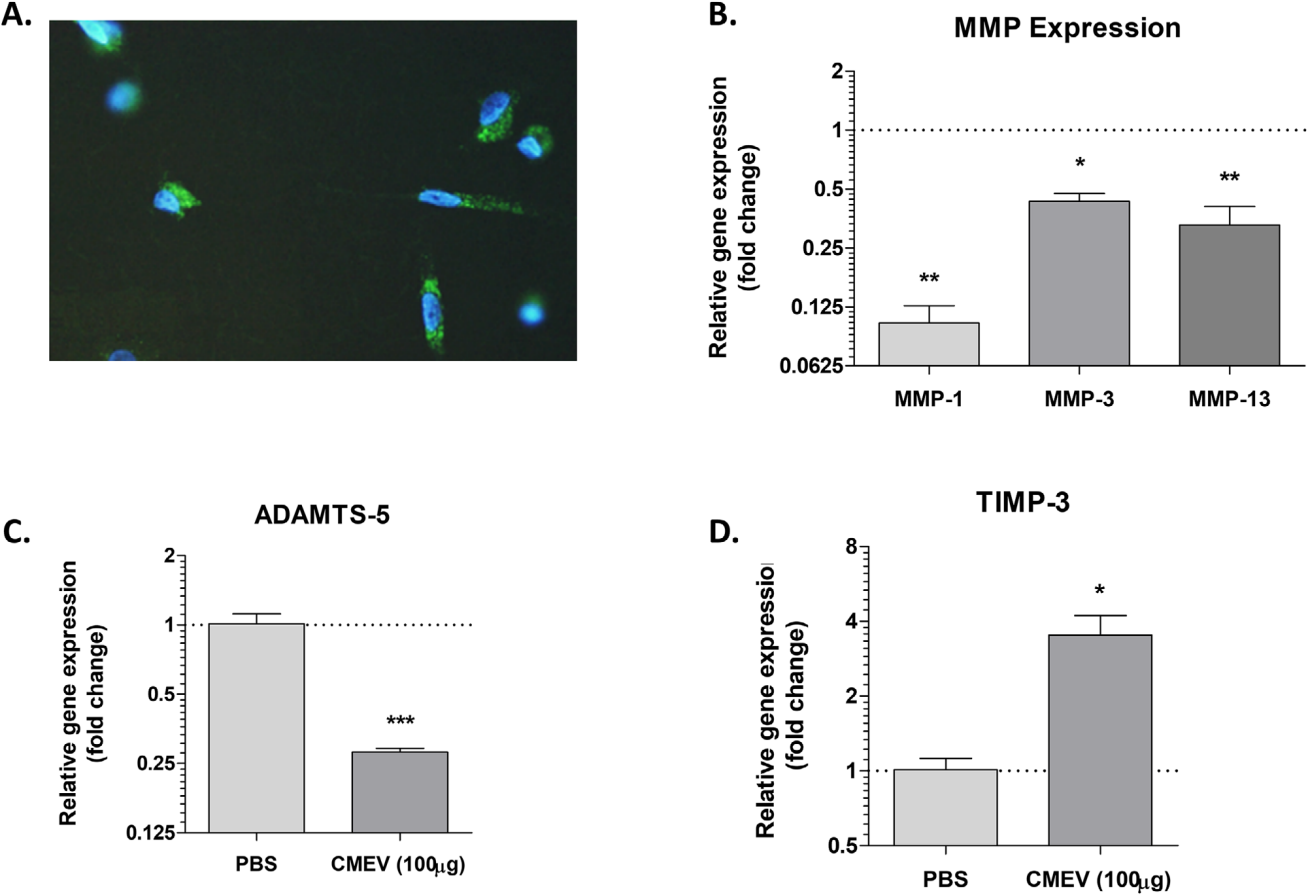
Milk‐derived EVs were taken up by chondrocytes and reduced catabolic gene expression in vitro. A) Chondrocytes in monolayer can take up fluorescently labelled (Green, PKH‐67) milk‐derived EVs after 24 h. Nuclei were stained using DAPI (Blue). B) 48‐h stimulation with CMEVs resulted in reduced MMP‐expression. C) *ADAMTS‐5* expression. D) *TIMP‐3* expression. Gene expression is plotted as fold change of CMEVs over PBS control. Data is shown from one representative experiment out of three individual experiments, performed in technical triplicates. Error bars represent mean ±SD, *p>0.05, **p>0.005, ***p>0.0005.

**Figure 4:**
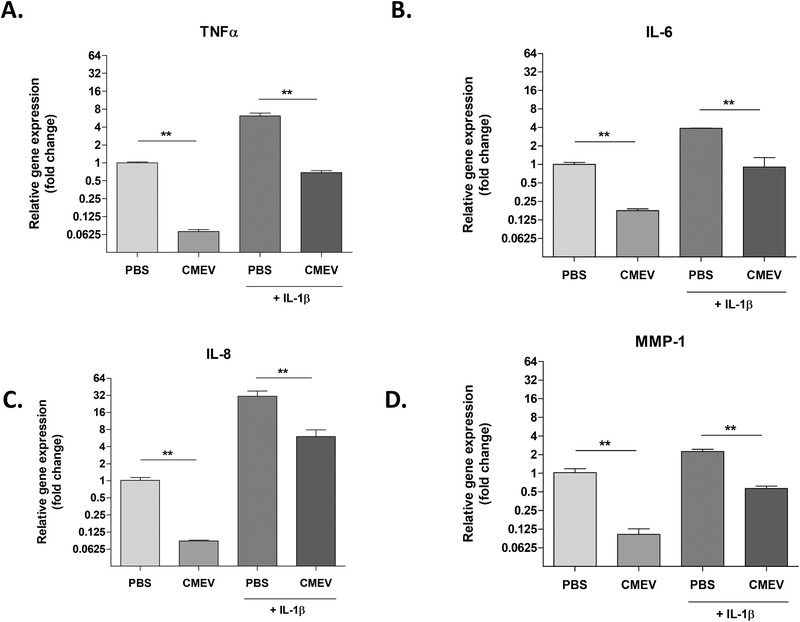
IL‐1β induced inflammatory response of chondrocytes was limited by milk‐derived EVs. Monolayer chondrocytes were stimulated with IL‐1β in the presence or absence of milk‐derived EVs (48hrs). IL‐1β stimulated chondrocytes showed an increased gene expression of (A) *TNFα*, (B) *IL‐6*, (C) *IL‐8*, and (D) *MMP‐1*. Meanwhile, CMEVs were able to reduce this expression both with and without IL‐1β stimulation present. Gene expression is plotted as fold change of CMEVs/IL‐1β over unstimulated PBS control. Error bars represent mean ±SD (*N* = 2 donors), *p>0.05, **p>0.005.

To study the mechanism behind the observed effect on OA cartilage in more detail, the G6 chondrocyte cell line was used. G6 cells are immortalized cells isolated from hip‐cartilage obtained from an OA patient.^[^ ^31^ ^]^ First, to determine if these chondrocytes could take up bovine milk‐derived EVs, fluorescently labeled EVs were used (method previously described in ^[^ ^17^ ^]^) (**Figure** **3**A). To ensure the staining was indeed vesicles being taken up, and not residual staining agent staining the cells or vesicles sticking to the membrane of the cells, a control was taken along which was incubated at 4°C, where no staining was observed. Thereafter the effects of bovine milk‐derived EVs on MMP‐ and ADAMTS‐expression were investigated. Similar to OA cartilage explants, a significant downregulation of *MMP‐1* (90%) was found, additionally reductions in *MMP‐3*, *MMP‐13*, and *ADAMTS‐5* expression (Figure 3B and 3C) were found. Lastly, the upregulation of *TIMP‐3* expression was confirmed, as shown in Figure 3D. Besides catabolic enzymes, inflammatory mediators contribute to the cartilage destruction observed in arthritic joint diseases. Interleukin‐1‐beta (IL‐1β) is thought to be one of the most important catabolic cytokines, and osteoarthritic chondrocytes have increased IL‐1β‐receptor expression compared to normal chondrocytes.^[^ ^34^ ^]^ To see if milk‐derived EVs could also limit catabolic enzyme expression in an inflammatory milieu, chondrocytes were stimulated with IL‐1β. Upon stimulation with IL‐1β, a strong induction of *TNFα, IL‐6, IL‐8*, and *MMP‐1* (**Figure** **4**) was observed. Milk‐derived EV stimulation resulted in a reduced expression of pro‐inflammatory cytokines and *MMP‐1*, in both conditions with and without IL‐1β present.

**Figure 5:**
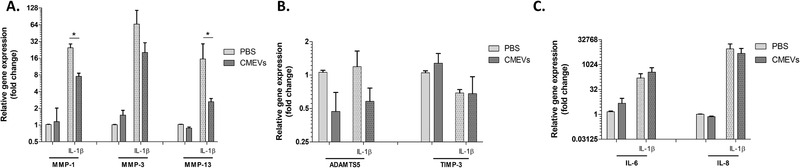
Diminished catabolic effects on primary chondrocytes by milk‐derived EVs. Primary chondrocytes were stimulated with (dotted bars) or without IL‐1β (clear bars) in the presence or absence of milk‐derived EVs (48 h). IL‐1β stimulated chondrocytes showed an increased gene expression of (A) MMP‐1, ‐3, and ‐13 after IL‐1‐β stimulation which are reduced by the added milk‐derived EVs (A). Also, reduction of ADAMTS5, both with and without IL‐1β stimulation was observed. No effects on TIMP‐3 were seen (B). Inhibition on the inflammatory responses of IL‐6 and IL‐8 were not seen, with or without IL‐1 β (C). Gene expression is plotted as fold change of CMEVs/IL‐1β over unstimulated PBS control. Error bars represent mean ±SD, *p>0.05, **p>0.005.

To validate our findings, freshly primary isolated human chondrocytes from two different donors were exposed to CMEVs in the presence or absence of IL‐1β (0.1 ng mL^–1^) for 48 h. The previous observed effects of EVs on the catabolic enzymes MMP‐1, MMP‐13, and MMP‐13 were confirmed (**Figure** **5**A). In addition, the mRNA levels of ADAMTS5 were reduced while effects on TIMP‐3 were not observed (Figure 5B). The previous mentioned effects on inflammatory mediators were not present (Figure 5C). The extreme low endogenic mRNA levels of these cytokines in these isolated human chondrocytes, probably due to the enzymatic isolation, seems a plausible explanation for this observation.

Altogether, this suggests that milk‐derived EVs can diminish cartilage degradation processes and potentially have an effect on inflammatory response as well, which could be beneficial in an inflamed and over‐catabolic environment, as seen in the cartilage of OA patients.

### 3.4 TGFβ is in Part Responsible for the Chondroprotective Effects of Milk‐Derived EVs

**Figure 6:**
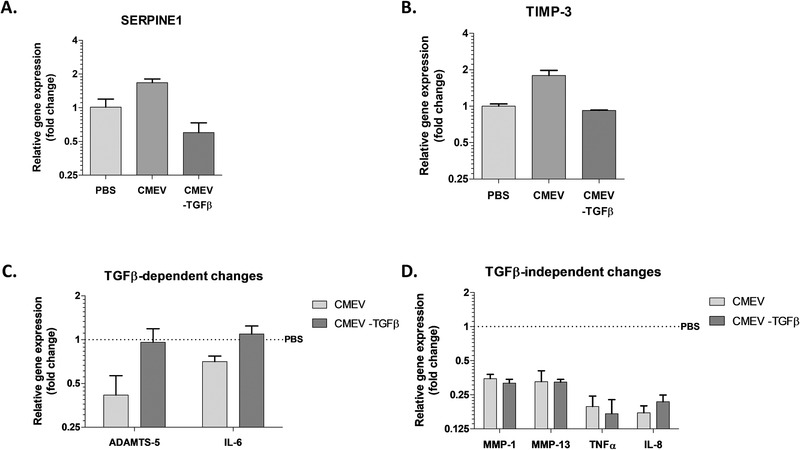
TGFβ mediated only part of the chondroprotective effects induced by milk‐derived EVs. Using an antibody‐based capture system, TGFβ‐positive CMEVs were separated from TGFβ‐negative CMEVs and used to stimulate chondrocytes in monolayer (48 h) in the absence of IL‐1β. A) Gene expression *SERPINE‐1*, (B) and TIMP‐3. C) *ADAMTS‐5* and *IL‐6* expression was no longer reduced using TGFβ‐negative CMEVs, whereas (D) *MMP‐1*, *MMP‐13*, *TNFα*, and *IL‐8* were not changed. Gene expression is plotted as fold change of CMEVs over PBS control (dashed line), performed in technical triplicates. Error bars represent mean ±SD.

It was previously shown by our group that commercial milk‐derived EVs contain active TGFβ, which is known to play dual role in the osteoarthritic joint. Under inflammatory conditions and at high concentrations, it seems to be deleterious for OA patients due to a shift in ALK1/5 expression, but at the same time, it controls repair and homeostasis at lower concentrations and in healthy cartilage.^[^ ^35^ ^]^ To better understand how milk‐derived EVs protect chondrocytes and cartilage, and whether active TGFβ plays a part in this process, an antibody‐based capture system to remove TGFβ‐expressing EVs was used. Using the capture ELISA, rather than complete blockade with an antibody, allowed us to determine the effects of vesicle‐bound TGFβ, instead of blocking all TGFβ, including paracrine production on which these cells depend during cultures. The analysis was focused on the same genes as shown before and included *SERPINE1* as positive control for TGFβ stimulation. As shown in **Figure** **6**A, expression of *SERPINE1* is induced by milk‐derived EVs, whereas TGFβ‐depleted EVs showed a reduction in *SERPINE1* expression. Similar to SERPINE1, induction of *TIMP‐3* by milk‐derived EVs was abolished without TGFβ‐positive EVs present (Figure 6B). Finally, *ADAMTS‐5* and *IL‐6* reduction was no longer observed without TGFβ‐positive EVs (Figure 6C), which is surprising as TGFβ alone showed a strong induction of both *ADAMTS‐5* and *IL‐6* expression in the chondrocyte (Figure S1, Supporting Information). *MMP‐1, MMP‐13, TNFα*, and *IL‐8* expression remained inhibited with and without the TGFβ present on the EVs (Figure 6D). These data suggest there is only a partial role for TGFβ in the chondroprotective effects with CMEVs observed in chondrocytes.

### 3.5 Milk‐derived EVs can Transfer Functional miR‐148a into Human Chondrocytes

**Figure 7:**
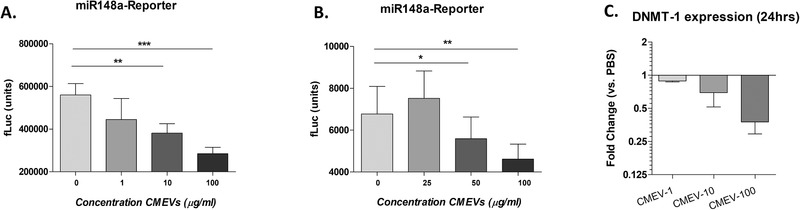
MiR‐148a was functionally transferred into chondrocytes and epithelial cells by milk‐derived EVs. Chondrocytes and A549 cells were transfected with a miR‐148a luciferase‐based reporter construct and stimulated with CMEVs for up to 72 h. Fluorescence was measured in chondrocytes (A) and A549 epithelial cells (B) exposed to different concentrations of CMEVs. C) Additionally, *DNMT‐1* expression was measured 24 h after stimulation. Error bars represent mean ±SD, **p>0.005, ***p>0.0005.

Besides active TGFβ, milk‐derived EVs are known to contain a high abundance of miRNAs.^[^ ^19^ ^]^ Among the highly expression miRNAs is miR‐148a, which is known to play a critical role in chondrocytes during OA. Vonk et al. have shown that miR‐148a expression is decreased in osteoarthritic cartilage and long‐term overexpression inhibited hypertrophic differentiation, with marked reductions in, among others, *MMP‐13* gene expression.^[^ ^36^ ^]^ To investigate whether miR‐148a is functionally transferred into chondrocytes via milk‐derived EVs, a luciferase‐based reporter construct was transfected into G6 chondrocytes.^[^ ^32^ ^]^ This reporter has a miR‐148a sequence in front of the 3’UTR of luciferase and is inhibited when miR‐148a binds. Upon stimulation with CMEVs a dose‐dependent reduction, at 10 and 100 µg mL^–1^, in luciferase activity was observed, indicative of functional inhibition of the reporter (**Figure** **7**A). This effect was also confirmed in another cell line, A549 cells (epithelial cells). These cells do not contain the SV‐40 construct, but showed a similar response, with efficient knockdown of the reporter (Figure 7B). Of note, the A549 cells were chosen since the basal level of miR‐148a is low in these cells.^[^ ^37^ ^]^ Additionally, the expression level for *DNMT‐1*, a well‐described direct target of miR‐148a,^[^ ^38^ ^]^ was measured. *DNMT‐1* expression was also reduced in a dose‐dependent manner after 24‐h stimulation (Figure 7C). Taken together, this confirms bovine milk‐derived EVs contain functional miR‐148a that can modulate target‐cell gene expression and could thereby contribute to the chondroprotective effects found.

## 4 Discussion

In this study, we investigated the functional effects of bovine milk‐derived EVs on human cartilage and cartilage‐resident chondrocytes. In our initial observation, we found that milk‐derived EVs can protect osteoarthritic cartilage from excessive glycosaminoglycan loss ex vivo. We identified a marked reduction in *MMP‐1* expression and upregulation of the MMP‐inhibitor *TIMP‐3* as potential underlying mechanisms. Furthermore, we found that chondrocytes are capable of taking up milk‐EVs and chondroprotective changes (reduction in catabolic and inflammatory mediators) were observed upon exposure to milk‐derived EVs. The functional transfer of miR‐148a is likely one of the mechanisms by which this protection occurs. This is the first demonstration of a functional effect for milk‐derived EVs on human chondrocytes and cartilage and highlights a therapeutic potential for these vesicles.

The association of dairy consumption and OA has long been under debate with conflicting study outcomes. The most recent Dutch observational study included 3010 participants and showed that there is no relation between milk consumption and presence of clinical knee OA.^[^ ^39^ ^]^ However, Lu et al. found that frequent milk consumption may be associated with reduced OA progression in women, quantified by a dose‐response relationship between milk intake and the decrease in joint space width over time.^[^ ^7^ ^]^ JSW is used as a measure for cartilage thickness in knees of OA patients and narrowing reflects cartilage degradation. We have previously shown that feeding mice bovine milk‐derived EVs in drinking water, comparable to daily consumption of two‐three glasses of milk for humans, is able to delay the onset of experimental arthritis and result in diminished cartilage pathology.^[^ ^25^ ^]^ In line with these results, here we show that in both human cartilage and monolayer chondrocytes bovine milk‐derived EVs can have a direct chondroprotective effect.

OA is characterized by degradation of articular cartilage, caused by an over‐catabolic and inflamed environment in the joint, in which chondrocytes play an important role.^[^ ^40^ ^]^ The collagenases, MMP‐1, and MMP‐13, have predominant roles in OA. In addition, MMP‐13 also degrades, aggrecan, giving it a dual role in matrix destruction. CMEVs were able to inhibit both of these enzymes, as well as increasing *TIMP‐3* expression in chondrocytes.

TGFβ is known to play a crucial role in the development of OA. In a healthy joint TGFβ is important for the maintenance of the cartilage, by keeping the chondrocytes in their differentiated phenotype.^[^ ^41^ ^]^ However, during OA TGFβ primarily signals via the Smad1/5/8 pathway, which has deleterious effects and drives chondrocytes into a hypertrophic state. Even though TGFβ is also known to inhibit IL‐1β signaling,^[^ ^42^ ^]^ CMEVs did not show this same inhibition. IL‐1β stimulation in the presence or absence of CMEVs led to a similar up‐regulation in *TNFα, IL‐6, IL‐8*, and *MMP‐1* gene expression. We therefore conclude that the anti‐catabolic and ‐inflammatory effect are not likely to be driven by TGFβ‐mediated alteration of IL‐1β signaling. Of note, there might be intravesicular TGFβ present in the milk EVs here described as TGFβ‐depleted, due to the use of an antibody‐based capture assay, however as the TGFβ receptor is present on the cell membrane, it is likely that only the active TGFβ on the outer milk vesicle membrane can interact with its receptor.

Previous studies on chondrocytes in OA have shown that has‐miR‐148a is expressed at lower levels in OA cartilage compared to healthy cartilage, and is suggested to play an important role in cartilage regeneration.^[^ ^36^ ^]^ Overexpression of miR‐148a in chondrocytes led to increased production of collagens, but also enhanced retention of proteoglycans in cartilage matrix. Similar to our observation using milk‐derived EVs, ADAMTS‐5 and MMP‐13 expression was decreased upon overexpression with miR‐148a. It is likely that this miRNA is central to the changes we observe in our chondrocytes and gives insight on how these bovine milk‐derived vesicles act. In addition to miR‐148, numerous other immune‐related miRNAs are expressed in milk EVs,^[^ ^17^, ^19^ ^]^ which could have beneficial effect to halt the progression of OA (reviewed in ^[^ ^43^ ^]^). In particular, miRNAs that can limit inflammation, such as miR‐21, 30a and 155, as this plays an important role in the pathogenesis of OA.

The inhibition of miR‐148a was observed using a single luciferase construct. We must attend that this inhibition could also be due to toxicity of the milk EVs, although a significant reduction of the miR148a reporter construct was observed even with 10 ug milk EVs. The upregulation of TIMP‐3 by 100ug milk EVs suggests that the used milk EV concentrations in this study were not toxic. The inhibition of DNMT‐1 expression, which is one of the target genes of miR‐148a, we report here is an interesting finding in the context of OA. Several studies have suggested epigenetic events play a large role in OA progression.^[^ ^44^ ^]^ Polymorphisms in the DNMT‐family members (DNMT‐1, DNMT‐3a, and DNMT‐3b) have also been found in relation to OA, with two polymorphisms in DNMT‐1 being associated with lower risk for primary knee OA.^[^ ^45^ ^]^ MiR‐148a also targets DNMT‐3a in chondrocytes and knockdown of DNMT‐3a results in inhibition of extracellular matrix degradation and chondrocyte apoptosis, which could be another pathway by which CMEVs function.^[^ ^46^, ^47^ ^]^

A limitation of our study could be the limited amount of different primary chondrocyte donors we used (*n* = 2). Additional donors would validate our observed findings and hopefully get a consensus of how the processes are affected by milk EVs. Furthermore, a potential pitfall in our study design is the use of damaged cartilage explants from end‐stage OA patients. Intact cartilage has an average pore size of 6.0 nm, which would mean milk EVs could not reach the embedded chondrocytes.^[^ ^48^ ^]^ However, as the material used in this study is of end‐stage OA patients, it is damaged and could allow for EVs to reach the exposed chondrocytes. In support of this idea, a recent study has shown that when cartilage is damaged (induced by IL‐1β stimulation), extracellular vesicles are able to penetrate the cartilage.^[^ ^49^ ^]^ The advantage could be that CMEVs only target chondrocytes that become exposed due to cartilage damage and not in unaffected areas. Important to note is that these chondroprotective effects have not yet been confirmed in vivo, and further studied into both the uptake via the digestive system (oral intake), as well as uptake and retention in the joint (intraarticular administration) should be performed.

The use of EVs in regenerative medicine, especially in tissue engineering, is a relatively new idea.^[^ ^50^ ^]^ The extracellular matrix (ECM) plays a major role in tissue engineering and EVs are able to influence ECM composition via direct interaction or by influencing ECM‐producing cells. The expression of adhesion molecules allows for example for the binding to collagen‐1 and fibronectin.^[^ ^51^ ^]^ An interesting example of this is the use of EVs obtained from pluripotent stem cells, for the treatment of osteoporotic ovariectomized rats. In the study by Qi et al., ^[^ ^52^ ^]^ they showed that these EVs can induce angiogenesis as well as osteogenesis, when incorporated on a porous scaffold, thereby promoting bone regeneration. The functionalization of such scaffolds, via addition of milk‐derived EVs, could be an alternative way of introducing the EVs into the joint. Rather than having to migrate from the place of absorbance, likely the gut, this would ensure the EVs end up in the affected joint. If milk‐derived EVs have similar ECM‐binding capacity remains to be determined, however, proteomic analysis of milk EVs have revealed the presence of similar adhesion molecules.^[^ ^53^ ^]^ Finally, in the study by Tao et al. miRNA‐140 overexpressing MSC‐derived EVs were directly injected in the articular cavity of rats, where they were able to prevent OA development. This further highlights the potential for locally administered EVs as potential therapeutics. The administration of EVs locally would also be preferred over oral administration in light of a recent publication by Samuel et al., ^[^ ^54^ ^]^ which highlights the potential adverse effects of orally administration of bovine milk‐derived EVs. Their manuscript shows that even though milk‐derived EVs can reduce the primary tumor burden, it also accelerates metastasis in a context‐dependent manner. They found that in the primary tumor milk EVs can induce senescence and epithelial‐mesenchymal transition, thereby reducing the primary tumor, but simultaneously accelerate the metastasis. This is an important finding, as OA is a disease primarily found in an aging population, which are at an increased risk of cancer development. Although a link between consumption of milk and cancer development has not been confirmed, the population studies thus far show conflicting results in a cancer‐type dependent matter. There are studies highlighting the increased risk of for example prostate cancer,^[^ ^55^ ^]^ but a consensus has not been reached on this yet. Nonetheless, in light of the recent publication by Samuel et al. the importance of further investigation in the safety of administering bovine milk‐derived EVs is evident.

In conclusion, milk‐derived extracellular vesicles can have a protective effect on cartilage by limiting both catabolic and inflammatory processes. A potential underlying mechanism is the supplementation of miR‐148a, which is functionally transferred into chondrocytes upon milk‐EV exposure. However, EVs are complex structures carrying a multitude of signals, and there are likely yet unknown mechanisms involved. Important to note that milk EVs can also promote bone regeneration and the inclusion of bone markers, it will therefore be important to find an optimal dose of milk EVs to achieve an equilibrium between cartilage protection and bone grow.

Milk‐derived extracellular vesicles could be injected locally or used to functionalize scaffolds, as a potential therapeutic for OA to reduce cartilage damage. That said, more research is warranted for showing the effect on bone and inflammation in OA and our results justify such efforts.

## Conflict of Interest

The authors declare no conflict of interest.

## Supporting information



Supplementary figure 1. TFGβ stimulated chondrocytes show an increased ADAMTS‐5 and IL‐6 gene expression.
Monolayer chondrocytes were stimulated with 0.1 ng mL–1 TFGβ . Chondrocytes showed an increased gene expression of *ADAMTS‐5* and *IL‐6* in response.

Supplementary table 1. MISEV2018 Checklist.

## Data Availability

The data that support the findings of this study are available from the corresponding author upon reasonable request.
