# Supplementary figures and images for: Bovine Milk‐Derived Extracellular Vesicles Inhibit Catabolic and Inflammatory Processes in Cartilage from Osteoarthritis Patients
Source: Mol Nutr Food Res. 2022 Jan 13;66(6):2100764. doi: 10.1002/mnfr.202100764 (PMC9285407; doi:10.1002/mnfr.202100764)

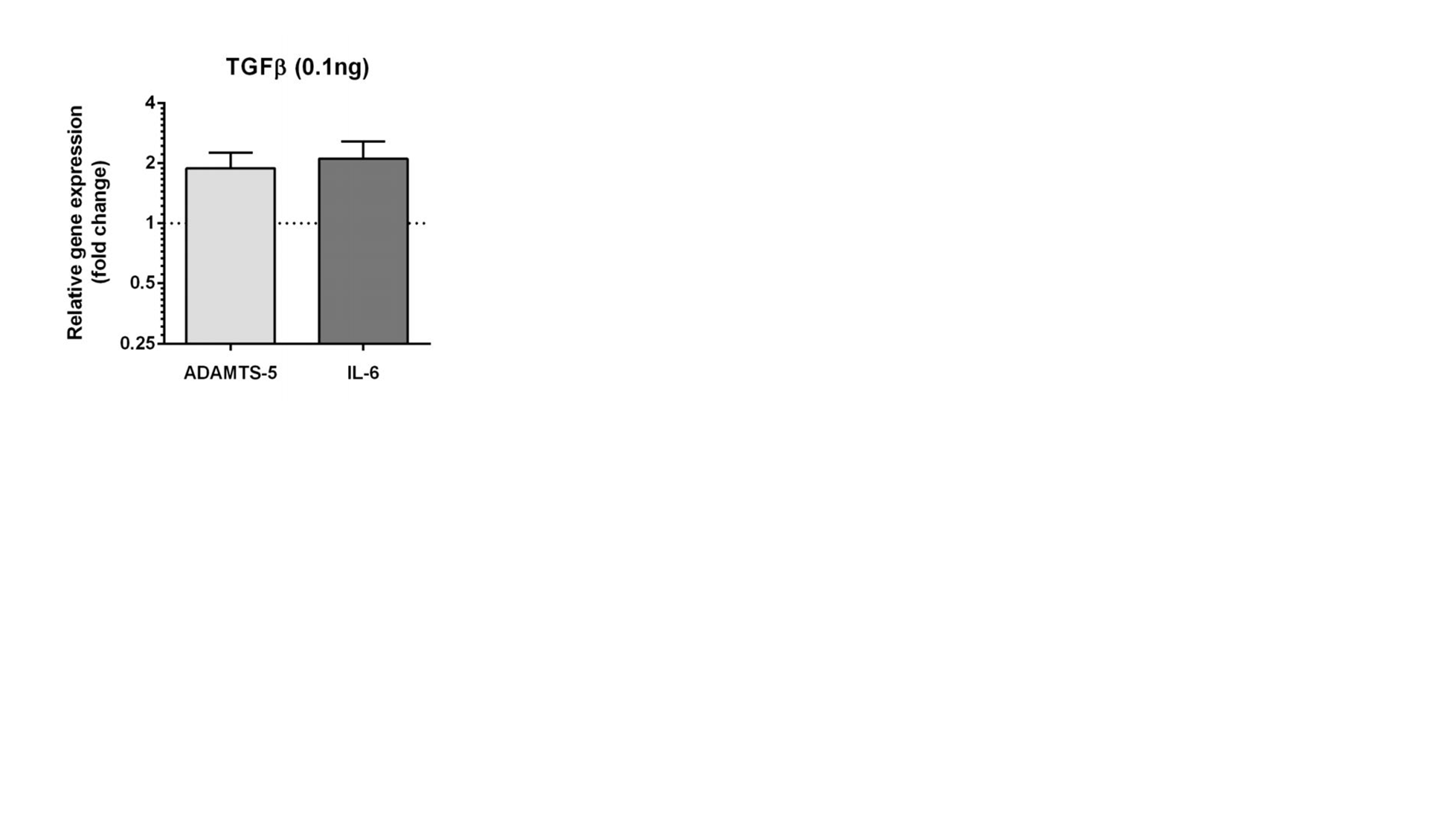

Supplement: Supplementary file 1 — Supplementary figure 1. TFGβ stimulated chondrocytes show an increased ADAMTS‐5 and IL‐6 gene expression. Monolayer chondrocytes were stimulated with 0.1 ng mL–1 TFGβ . Chondrocytes showed an increased gene expression of ADAMTS‐5 and IL‐6 in response. [file MNFR-66-0-s001.tif]

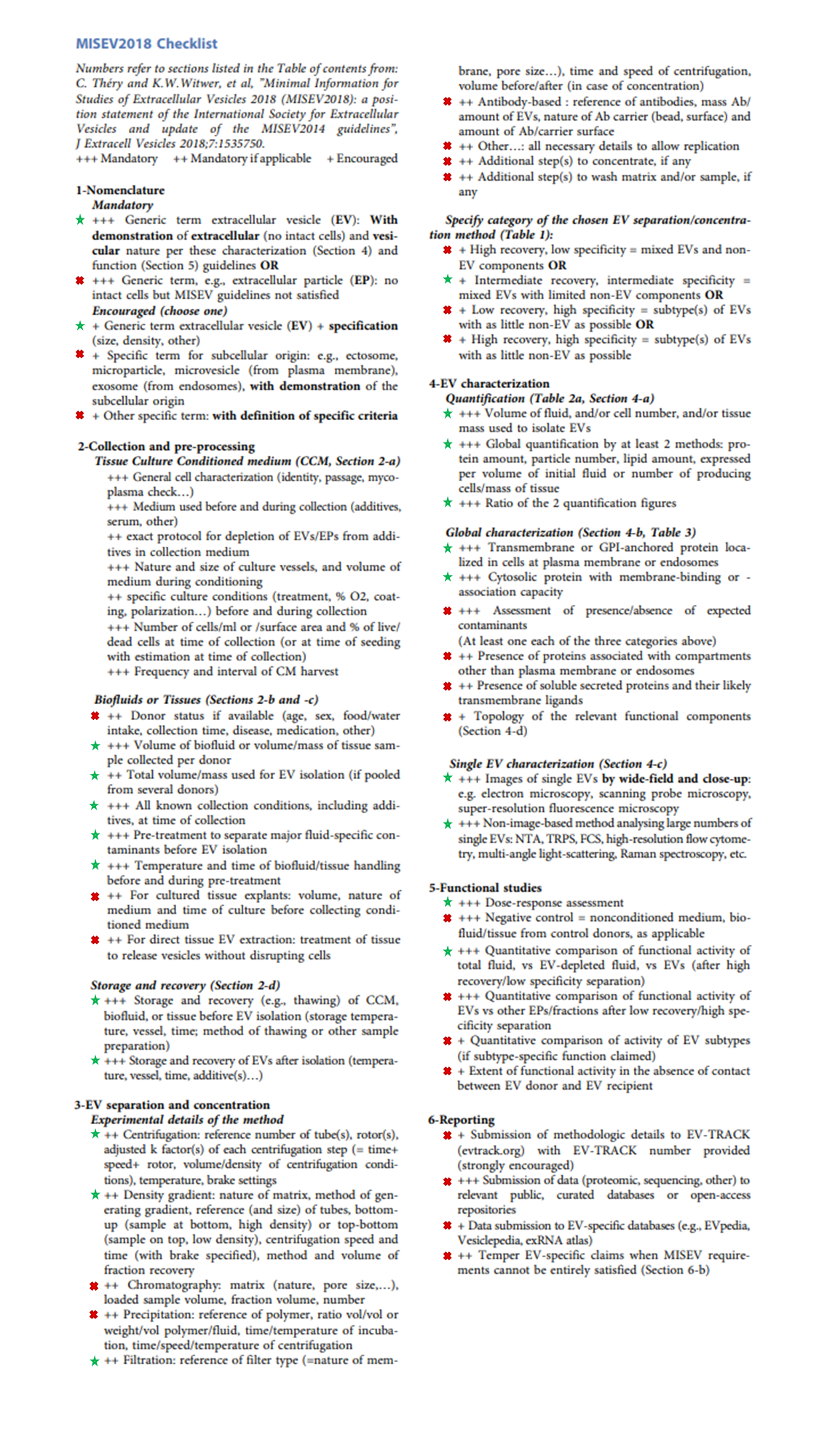

Supplement: Supplementary file 2 — Supplementary table 1. MISEV2018 Checklist. [file MNFR-66-0-s002.tif]
